# Nephrotoxicity and Genotoxicity of Silver Nanoparticles in Juvenile Rats and Possible Mechanisms of Action

**DOI:** 10.2478/aiht-2020-71-3364

**Published:** 2020-06-29

**Authors:** Ye Liu, Li Sun, Guili Yang, Zhuo Yang

**Affiliations:** 1 Tianjin Medical University General Hospital, Ministry of Education and Tianjin City, Tianjin Neurological Institute, Key Laboratory of Post-Neuroinjury Repair and Regeneration in Central Nervous System, Tianjin, China; 2 Nankai University College of Medicine, State Key Laboratory of Medicinal Chemical Biology, Key Laboratory of Bioactive Materials for Ministry of Education, Tianjin, China

**Keywords:** alkaline comet assay, inflammation, oxidative stress, trpc6 cation channel, alkalni komet test, oksidacijski stres, TRPC6 kationski kanal, upala

## Abstract

Because of their widespread use and potential adverse effects in young developing organism, this study focused on the nephrotoxicity and genotoxicity of chronic low-dose exposure to silver nanoparticles (AgNPs) in 32 14-day-old male Wistar rats, randomly divided into three groups receiving AgNP solution (3 mg/kg body weight) intraperitoneally for one, two, or three weeks and the untreated control group (eight animals per group). When the rats were eight weeks old, blood creatinine and urine microalbumin were tested, followed by haematoxylin and eosin (H&E) staining. Proteinuria was found in the animals treated with AgNP for three weeks, and H&E staining revealed pathological changes in the kidney sections of this group. DNA damage was detected with the alkaline comet assay in the groups treated for two and three weeks. All results indicate that chronic exposure, even at a low dose, may affect animal health. The main culprit might be increased and time-dependent reactive oxygen species (ROS) production. Highly reactive ROS could cause a major structural damage to proteins and DNA, change the expression of ion channel proteins, and trigger inflammation. The findings of our *in vivo* experiment raise concern about nephrotoxic and genotoxic effects of silver nanoparticles in young organisms and call for further investigation of nanoparticle properties that can be modified to minimise the risks.

## Abstract (HR)

Zbog široke primjene i rizika od štetnog djelovanja srebrnih nanočestica (AgNP) na mladi organizam u razvoju, u središtu pažnje ovog istraživanja bila je njihova nefrotoksičnost i genotoksičnost u 32 mužjaka Wistar štakora od 14. dana starosti koji su intraperitonealno primali otopinu AgNP-a (3 mg/kg tjelesne težine). Štakori su nasumce raspoređeni u tri izložene skupine (osam po skupini) – prva jedan tjedan, druga dva, a treća tri – i jednu kontrolnu skupinu, koja je primala fiziološku otopinu. Kad su štakori navršili osam tjedana, napravili smo pretrage krvi na kreatinin i mokraće na mikro albumin, a zatim i histološku pretragu s bojenjem hematoksilinom i eozinom (H&E). U životinja koje su tri tjedna bile izložene AgNP-u utvrđena je proteinurija, a H&E bojenjem utvrđene su patološke promjene na presjecima bubrežnog tkiva. Alkalnim komet testom otkriveno je oštećenje DNA u skupinama izloženima dva odnosno tri tjedna. Svi naši rezultati upućuju na to da kronična izloženost AgNP-u, makar i u niskim dozama, može utjecati na zdravlje životinja. Primarni je razlog za to vjerojatno povišena proizvodnja reaktivnih kisikovih spojeva (engl. reactive oxygen species, krat. ROS), koja je rasla s vremenom izloženosti. Visokoreaktivni kisikovi spojevi mogu uzrokovati velika strukturna oštećenja bjelančevina i DNA, izmijeniti ekspresiju ionskih kanala bjelančevina te potaknuti upalu. Rezultati našega in vivo istraživanja pozivaju ne samo na zabrinutost zbog nefrotoksičnih i genotoksičnih učinaka srebrnih nanočestica na mlade organizme nego i na potrebu daljnjeg istraživanja promjenjivih svojstava nanočestica ne bi li se rizik od njihova štetnog djelovanja sveo na najmanju moguću razinu.

Nanoparticles are materials whose at least one dimension is within the 1–100 nm range. As their size determines their physicochemical properties and can give them an edge over or complement standard chemicals (1), nanomaterials are being rapidly developed for a wide range of uses in all industrial and public sectors. One such material are silver nanoparticles (AgNPs), whose broad-spectrum antimicrobial properties find use in medicinal and everyday products such as diapers, baby bottles, and baby wipes (2, 3).

However, some animal studies report that silver ions can accumulate in body organs and that AgNPs can have toxic effects on the liver and kidney (4, 5). Considering that the kidneys in a young, developing organism are much more sensitive to exogenous compounds than those in a mature system, it is important to assess the potential of AgNPs to cause developmental nephrotoxicity and genotoxicity. This is the primary aim of this study in juvenile Wistar rats, as this issue has received little attention so far.

The secondary aim was to see whether the main cause of these effects is oxidative stress and its downstream pathways. Having a larger surface area, nanoparticles may induce higher free radical and reactive oxygen species (ROS) production than their non-nanoform chemical counterparts (6, 7). Earlier research suggests that the major enzymatic source of ROS in AgNP-induced cytotoxicity is nicotinamide adenine dinucleotide phosphate (NADPH) oxidase, also known as NOX (8). Its isoform NOX4 is highly expressed in the kidney and has an important role in kidney diseases (9, 10). Changes in NOX4 levels may reflect on ROS production, and excessive production can lead to oxidative stress and inflammation that can eventually lead to the development of chronic kidney disease (11).

ROS effects on cellular processes are mediated by multiple downstream pathways, and the Ca^2+^ signalling pathway is perhaps the most important, because Ca^2+^ cell homeostasis is redox-sensitive (12, 13, 14, 15), and the Ca^2+^-permeable transient receptor potential cation channel 6 (TRPC6) is widely expressed in kidney cells, including podocytes (16), glomerular mesangial cells (17), and endothelial cells (18). Animal studies have showed that podocyte-specific transgenic TRPC6 overexpression can lead to albuminuria and histological findings similar to human focal segmental glomerulosclerosis (FSGS) (19). Wang et al. (20) have already shown that TRPC6 is redox-sensitive and suggested that ROS could regulate Ca^2+^ signalling by altering TRPC6 protein expression or TRPC6 channel activity in kidney cells.

In addition, nanoparticles may elicit genotoxic effects, as ROS can react with the DNA (21), and evidence of *in vitro* and *in vivo* genotoxic effects of AgNPs is growing (22, 23).

## Materials and methods

### Materials

**Figure 1:**
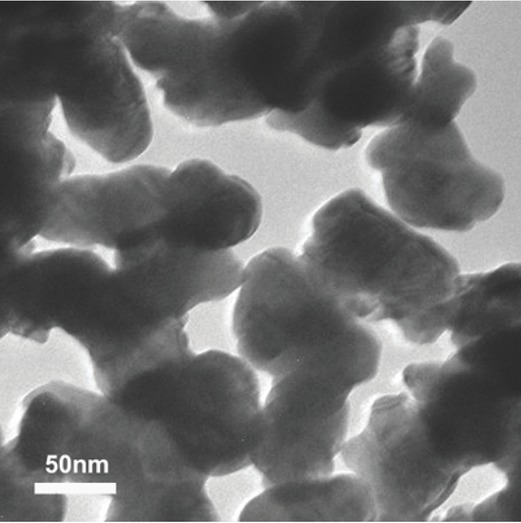
Transition electron microscopy of silver nanoparticles in suspension

The silver nanoparticles (Figure 1) used in this research were obtained from the University of Hertfordshire, England, UK. They were suspended in deionised water at a concentration of 1 mg/mL and dispersed with ultrasonic vibration for 20 min before everyday injection. The mean hydrodynamic particle size in suspension was 98.3 nm (range 30.3–178.1 nm due to aggregation) as measured with dynamic light scattering (DLS) using a Zeta-PALS+BI-90 Plus (Brookhaven Instruments Corporation, Hotsville, NY, USA) at a wave length of 659 nm and the scattering angle of 90 °. Surface area, measured with the Brunauer Emmett and Teller (24) method was 114 m^2^/g. The Zeta potential of -36.58 was measured in the suspension with a combination of laser Doppler velocimetry and phase analysis light scattering (PALS) using Zeta-PALS+BI-90 Plus.

As for other materials we used the reactive oxygen species assay kit (Nanjing Jiancheng, Nanjing, China), rabbit polyclonal anti-TRPC6 antibodies, rabbit polyclonal anti-NADPH oxidase 4 (primary antibody) (Abcam, Cambridge, MA, USA), Alexa 488-conjugated goat anti-rabbit IgG antibodies (secondary antibody, working dilution 1:1000) (Invitrogen, San Diego, CA, USA), rabbit polyclonal anti-β-actin IgG (primary antibody, working dilution 1:1000) (Santa Cruz Biotechnology, Inc., Santa Cruz, CA, USA), chemiluminescent HRP substrate (Immobilon Western, Millipore Corporation, Billerica, MA, USA), haematoxylin and eosin (H&E) staining kit (Solarbio, Beijing, China), creatinine (Cr) assay kit (Nanjing Jiancheng), microalbumin assay kit (Nanjing Jiancheng), total protein assay kit (Nanjing Jiancheng), enzyme-linked immunosorbent assay (ELISA) kit for nicotinamide adenine dinucleotide phosphate oxidase 4 (NOX4) (Shanghai Enzyme-linked Biotechnology Co., Ltd. Shanghai, China), rat IL-6 ELISA kit and rat TNF-α ELISA kit (NeoBioscience, Guangdong, China), rat IL-1β ELISA kit (BioGems, New Jersey, NJ, USA), GelRed^TM^ nucleic acid gel stain (Biotium, San Francisco, CA, USA), and radioimmunoprecipitation assay (RIPA) lysis buffer (Beyotime, Shanghai, China).

### Animals and AgNP treatment

**Figure 2:**
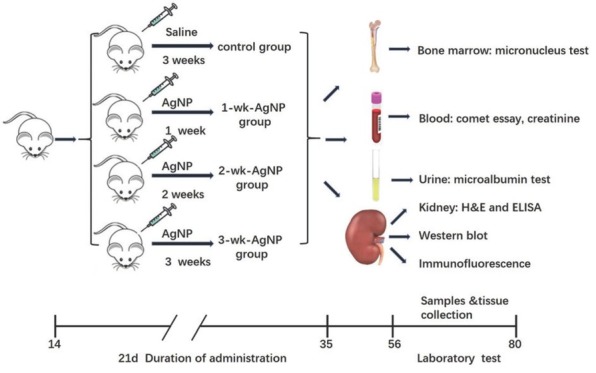
Experimental design of AgNP administration, sampling, and tissue collection; AgNP – silver nanoparticles; H&E – haematoxylin and eosin; ELISA – enzyme-linked immunosorbent assay

Figure 2 shows the experimental design for this study, in which we used 32 Wistar male rats. The pups were housed with the dam and their litter until weaning on postnatal day 21, after which they were randomly assigned to either the control or one of the three treatment groups, eight to each, and moved to respective cages. All three treatment groups received intraperitoneal injection of AgNP solution with 3 mg of AgNP per kg of body weight (bw) every day. This dose was based on our previous findings of its effects (lower doses showed none) (7) and preliminary analysis. The first group was treated with AgNPs for one week (1-wk-AgNP), the second for two weeks (2-wk-AgNP), and the third for three weeks (3-wk-AgNP). The control group received sterile saline alone.

We observed specific pathogen-free conditions throughout the experiment. The environmental conditions were kept at 22±1 °C and 45–55 % relative humidity, with a 12:12 h light/dark cycle. Clean water and conventional diet were provided *ad libitum*.

When the rats reached adulthood at eight weeks, they were killed under isoflurane anaesthesia and their blood and urine samples collected and kidneys removed.

All the protocols were approved by the Committee for Animal Care of Tianjin Medical University. All animal experiments complied with the EU Directive 2010/63/EU (25). The experiment was designed to minimise the number of animals used and their suffering.

### Creatinine and urinary microalbumin detection

Depending on the treatment group, the animals were given time to recover from AgNP exposure until they reached adulthood at 8 weeks. Overnight urine samples were collected in metabolic cages and blood samples drawn from the tail vein with a syringe. Urinary microalbumin was detected with the microalbumin assay kit. Serum was harvested after blood centrifugation at 1902 ×*g* for 10 min and creatinine measured with the creatinine assay kit. All procedures were performed following the kit instructions.

### Alkaline comet assay

The micronucleus and the single-cell gel electrophoresis (comet) are the most common tests used *in vitro* or *in vivo* to investigate genotoxicity of nanoparticles (26, 27, 28). We performed the comet assay as described by Tice et al. (29) and Machado et al. (30). Cell viability was above 90 %, according to the trypan blue dye exclusion method. Samples of peripheral blood were immediately mixed with 0.5 % of low melting point agarose dissolved in phosphate-buffered saline (PBS) and spread on microscope slides precoated with 1.5 % (w/v) of normal melting point agarose. After solidification of the gel (within about an hour), the coverslips were gently removed and the slides immersed in cold (4 °C) lysis solution (2.5 mol/L NaCl, 100 mmol/L EDTA, 1 % (v/v) triton X-100, and 10 mmol/L Tris at pH 10) for 24 h. After that, the slides were placed in a horizontal electrophoresis unit containing freshly prepared electrophoresis buffer (300 mmol/L NaOH and 1 mmol/L EDTA) for 20 min at an electric field strength of 0.78 V/cm (25 V and 300 mA). When electrophoresis was over, the slides were immersed in a neutralisation buffer (0.4 mol/L Tris–HCl, pH 7.5) for 5 min and stained with GelRed^TM^ (1:10,000). All slides were randomly coded for five animals per group. For each slide (two per rat) we scored 50 cells using the Comet Assay IV^®^ software (Perceptive Instruments Ltd., Suffolk, UK), and determined tail intensity (% DNA in tail) as the genotoxicity parameter.

### Bone marrow cell micronucleus test

The frequency of micronuclei is a characteristic parameter indicative of chromosomal loss and breakage (20). Bone marrow cells of five animals per group were harvested following the method described by Salamone et al. (31) and the micronucleus test performed as described by Schmid (32). After being homogenised and centrifuged at 400×*g* for 10 min, the cells were resuspended and layered on a microscopic slide. The slides were randomly coded, fixed in absolute methanol for 10 min, and stained with Giemsa. Two slides were prepared per animal. Two thousand polychromatic erythrocytes (PCE) were scored per animal (10,000 per group) for the occurrence of micronuclei. Normochromatic erythrocytes (NCE) were counted in parallel to calculate the PCE to NCE ratio.

### Kidney ROS, glutathione, and superoxide dismutase assays

After blood and urine sample collection, the rats were killed and their kidneys removed and prepared for single-cell suspensions. Half of the suspension volume was used to determine the levels of ROS, glutathione (GSH), and superoxide dismutase (SOD) following kit instructions. The other half was used to determine protein concentrations.

### Inflammatory cytokine determination

Inflammatory cytokines (IL-1β, IL-6, and TNF-α) were determined in kidney tissue homogenates with the ELISA kits following kit instructions.

### Western blotting

Renal cortex was isolated, minced into fragments, and lysed in a lysis buffer containing a protease inhibitor cocktail (1:1000 dilutions). After preparation of protein extracts, western blotting was conducted as described elsewhere (33). For the analysis, we quantified the intensity of hybridisation signals. Image J program (https://imagej.nih.gov/ij/) was used to evaluate differences between the samples of interest and respective β-actin.

### Immunofluorescence staining

Kidney samples were embedded in the optimum cutting temperature (OCT) formulation (Tissue-Tek, Torrance, CA, USA) after being fixed in 4 % paraformaldehyde for 24 h and dehydrated in 30 % sucrose solution at 4 °C overnight. Seven micron thick slices were cut with a freezing microtome (Leica CM 1850, Leica Microsystems Nussloch, Wetzlar, Germany) and washed in PBS three times for 5 min and then incubated in a blocking buffer with 10 % goat serum for 1 h. After that, the slices were incubated in primary antibody (anti-TRPC6, 1:1000) at 4 °C overnight. Sections were protected from light, washed in PBS three times for 10 min to get rid of non-specific binding, and incubated in Alexa 488-conjugated anti-rabbit IgG. When all the steps were finished, fluorescent images were inspected with a Leica TCS SP5 laser-scanning confocal microscope (40x magnification). Image-Pro Plus software (Version VI; Media Cybernetics, Silver Springs, MD, USA) was used to quantify intensity in the renal slices. For TRPC6 expression analysis, three sections were selected randomly from one kidney sample, and there were eight samples for each group.

### Histology

Rat kidneys for histological analysis were removed carefully and fixed in a 10 % formalin solution containing neutral PBS. After dehydration with graded ethanol and vitrification with dimethylbenzene, the organs were embedded in paraffin, cut into slices, H&E-stained following the kit instructions, and examined under a light microscope (40x magnification, Nikon, Tokyo, Japan).

### Statistical analysis

Means ± standard errors (SEM) of all data were analysed using one-way analysis of variance (ANOVA) followed by a Newman-Keuls *post hoc* test. Animal body weights were analysed with two-way ANOVA with groups as between-subject factor and time as repeated measure. All analyses were run with statistical software SPSS v. 17.0. (IBM, Armonk, NY, USA). *P*<0.05 was considered significant.

## Results

### Physical findings

**Figure 3:**
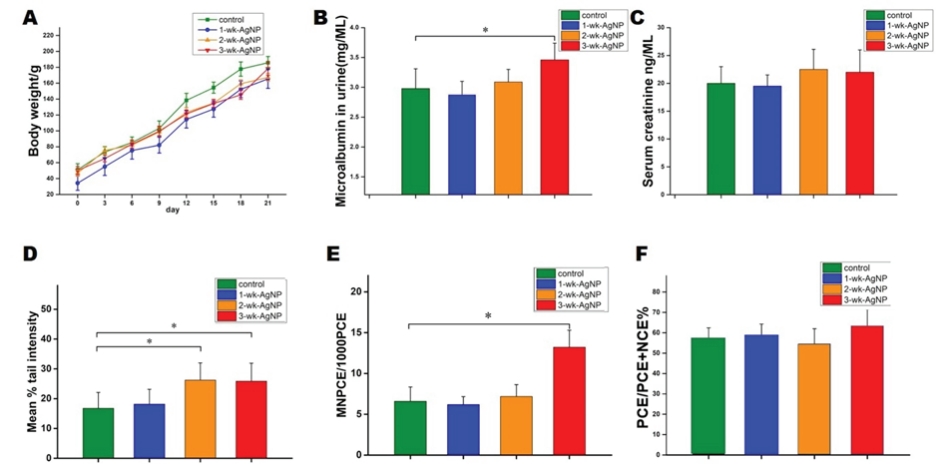
Mean (±SEM) body weight (A), urinary microalbumin (B), serum creatinine (C), tail intensity (comet assay) (D), the frequency of micronucleated PCEs in bone marrow (E), and the PCE to NCE ratio (F) in control and juvenile rats treated with AgNPs for one, two, or three weeks (n=8 per group); *P<0.05; AgNP – silver nanoparticles; NCE – normochromatic erythrocytes; PCE – polychromatic erythrocytes; SEM – standard error of the mean

No mortality related to AgNP administration was observed during this study. There were no significant differences in body weight between the control and AgNP-treated groups (Figure 3A).

### Urinary microalbumin and serum creatinine findings

Urinalysis results are shown in Figure 3B. Urinary microalbumin was significantly higher in the 3-wk-AgNP group than controls [F(3, 28)=3.83, *P<*0.05], but it did not differ significantly from the other two treatment groups. Serum creatinine showed no statistical differences between any of the four groups, although it was slightly higher in the 2- and 3-wk-AgNP group than controls [F(3, 28)=1.67, *P>*0.05] (Figure 3C).

### Alkaline comet assay findings

Significantly higher tail intensity in the 2- and 3-wk-AgNP groups than controls [F(3, 28)=3.07, *P<*0.05] suggests that the DNA damage depended on the length of nanoparticle exposure.

### Micronucleus test findings

Figure 3E shows the frequency of micronucleated PCEs in bone marrow, while Figure 3F shows the PCE to NCE ratio. The 3-wk-AgNP group had a significantly higher micronucleus frequency than controls [F(3,28)=3.18, *P*<0.05], but there was no statistical difference in the PCE to NCE ratio between any of the groups [F(3,28)=2.32, *P*>0.05].

### Kidney ROS, GSH, and SOD findings

**Figure 4:**
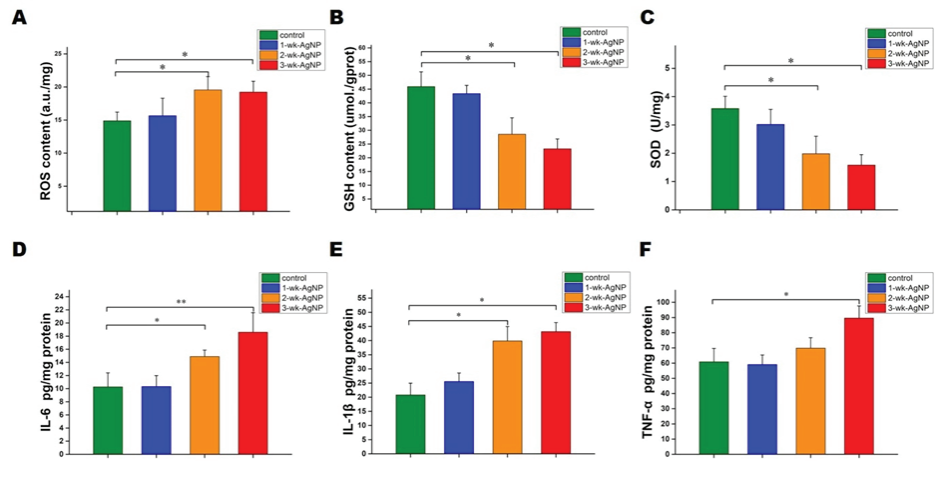
Mean (±SEM) ROS GSH, SOD IL-6, IL-1β, and TNF-α in kidney cells and tissue of control and juvenile rats treated with AgNPs for one, two, or three weeks (n=8 per group); *P<0.05; GSH – glutathione; IL – interleukin; ROS – reactive oxygen species; SEM – standard error of the mean; SOD – superoxide dismutase; TNF – tumour necrosis factor

The 2- and 3-wk-AgNP groups had significantly higher ROS [F(3,28)=3.31, *P*<0.05] (Figure 4A) and significantly lower GSH and SOD levels in the kidney tissue than control [F(3,28)=3.36, *P*<0.05] (Figure 4B and 4C).

### Inflammatory cytokine findings

Figure 4D–F shows inflammatory cytokine levels in the four groups. As expected, IL-6 and IL-1β were significantly higher in the 2- and 3-wk-AgNP groups than control [F(3,28)=3.72 and 3.28, respectively, *P*<0.05] (Figure 4D and 4E), while the level of TNF-α was significantly higher only in the 3-wk-AgNP group [F(3,28)=3.16, *P*<0.05] (Figure 4F). We also observed a coincidence in trends between inflammatory cytokines and ROS levels, which suggests a link between inflammation and oxidative stress.

### Western blot findings

**Figure 5:**
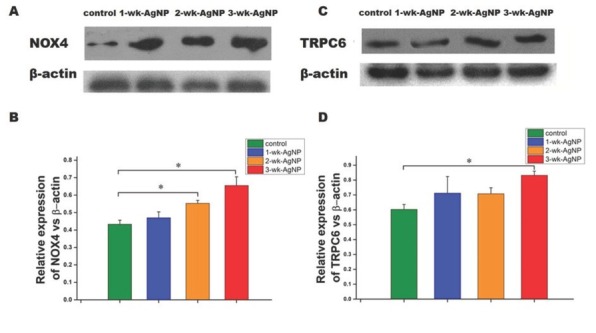
Mean (±SEM) NOX4 and TRPC6 in rat renal cortex (normalised to β-actin expression) of control and juvenile rats treated with AgNPs for one, two, or three weeks (n=8 per group); *P<0.05; NOX – NADPH oxidase 4; SEM – standard error of the mean; TRPC6 – transient receptor potential cation channel subfamily C member 6

The 2- and 3-wk-AgNP groups showed significantly higher expression of NOX4 than controls [F(3,28)=3.09, *P*<0.05] (Figure 5B), which suggests that higher ROS production was at least partly owed to the mobilisation of NOX4. At the same time, TRPC6 expression was significantly higher only in the 3-wk-AgNP group [F(3,28)=2.98, *P*<0.05] (Figure 5D).

### Immunofluorescence staining findings

**Figure 6:**
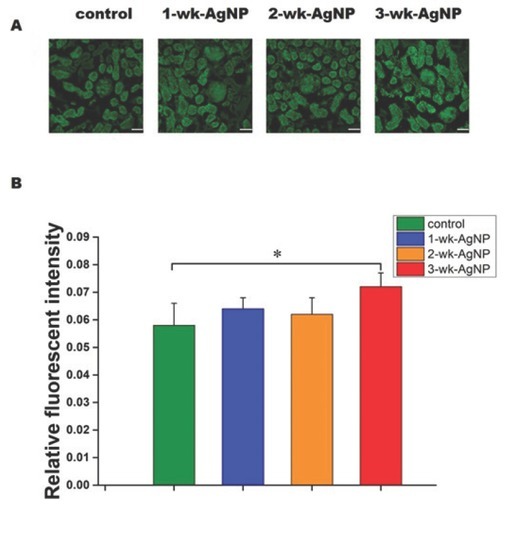
TRPC6 immunofluorescence staining of the renal cortex (50 μm scale bar) (A) and mean (±SEM) fluorescent intensity (B) in the glomeruli of control and juvenile rats treated with AgNPs for one, two, or three weeks (n=8 per group); *P<0.05; SEM – standard error of the mean; TRPC6 – transient receptor potential cation channel subfamily C member 6

Figure 6A shows TRPC6 in the glomeruli and tubules of the kidney cortex and its fluorescent intensity (Figure 6B), which was significantly higher only in the 3 -wk-AgNP group compared to control [F(3,28)=3.23, *P*<0.05].

### H&E staining findings

**Figure 7:**
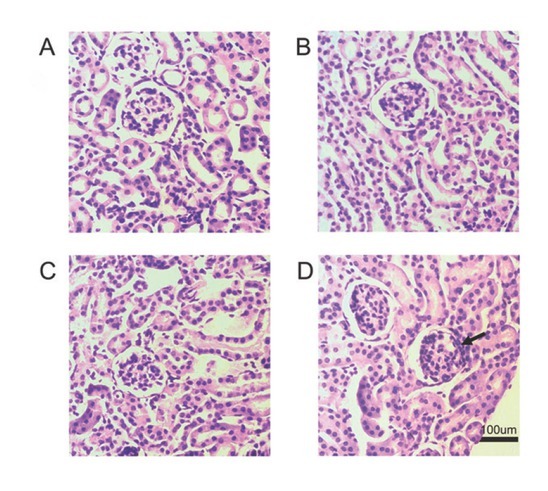
Histological findings in the kidney of juvenile rats: (A) control group, (B) 1-wk-AgNP group (C) 2-wk- AgNP group; (D) 3-wk-AgNP group (100 μm scale bar; arrows indicate typically affected areas)

H&E staining revealed pathological changes of several glomeruli in the 3-wk-AgNP group, which included glomerular cell proliferation, thickened basement membrane, fusion of glomerulus and renal capsule, and proliferation in the mesangial cell and mesangial matrix. The interstitial areas were infiltrated with inflammatory cells. Renal tubules did not show pathological changes (Figure 7).

## Discussion

**Figure 8:**
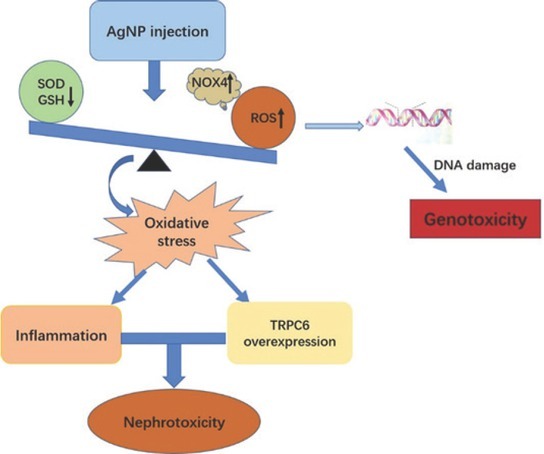
Diagram of the main likely mechanisms of AgNP nephrotoxicity based on oxidative stress; AgNP – silver nanoparticles; H&E – haematoxylin and eosin; ELISA – enzyme-linked immunosorbent assay; GSH – glutathione; ROS – reactive oxygen species; SOD – superoxide dismutase; TRPC6– transient receptor potential cation channel subfamily C member 6

Our study suggests that chronic exposure to silver nanoparticles can cause DNA damage, inflammation, and TRPC6 overexpression due to oxidative stress, which, in turn, may lead to nephrotoxicity and genotoxicity (Figure 8). Our findings also suggest that these effects are related to exposure duration. Unlike the *in vivo* study by Sarhan et al. (34), in which an acute AgNP dose of 2,000 mg/kg body weight induced significant structural and functional liver and kidney damage, we observed only mild functional damage and pathological changes in the kidneys of our juvenile rats. However, even our small dose (3 mg/kg body weight), which reflects real-life daily exposure to AgNPs through products such as diapers, baby bottles, or nanosilver baby wipes, suggests that frequent exposure may affect human health if it exceeds a toxicological threshold.

The *in vivo* comet assay and micronucleus test only confirmed time-dependent (genotoxic and chromosomal damage) effects, which were the most prominent after three weeks of exposure.

What could be the likely mechanisms of nephrotoxicity and genotoxicity of AgNPs? Our previous study showed that AgNPs can cause brain damage owing to excessive generation of ROS (7). With significantly higher ROS levels after two and three weeks of exposure, this study seems to confirm oxidative stress as one likely mechanism. Furthermore, we observed a coincidence between DNA damage and increased ROS, accompanied by a significant decline in antioxidant (SOD) and GSH levels. This may have caused an oxidative imbalance that has given rise to oxidative stress.

Findings that silver nanoparticles induce oxidative stress by generating excess ROS have already been reported for the hepatoma cells *in vitro* (35). Highly reactive ROS seems to be generated by accumulated AgNPs in mitochondria and lysosomes, which then leads to major structural damage to proteins and DNA (36, 37). According to another study (10), the NOX family, NOX4 in particular, is the major source of renal ROS. Our study evidences a consistency between up-regulated NOX4 and ROS production, which points to NOX4 as the main source of AgNP-induced toxicity.

One of the oxidative stress pathways is inflammation. It has been reported in the pathogenesis of chronic kidney disease (11), and excessive ROS has been reported to activate mediator signalling molecules which trigger the production of inflammatory cytokines such as IL-1β or TNF-α (38, 39). The upregulated expression of TNF-α, IL-1β, and IL-6 after two- and three-week exposure to AgNPs in our study confirms the link between proinflammatory cytokines and ROS and suggests that excessive ROS production might play a part in AgNP-induced nephrotoxicity.

In addition, a number of studies (12, 13, 14, 15) have confirmed that Ca^2+^ homeostatic proteins are redox-sensitive, which makes the Ca^2+^ signalling pathway an important target of ROS. One such widely expressed Ca^2+^ channel in the kidney is the slit diaphragm-associated channel TRPC6 involved in regulating glomerular filtration, whose failure is closely related to proteinuria (40). Apparently, overexpression of TRPC6 causes retraction and loss of podocyte foot processes, which are responsible for filtration (41). Our study has shown exactly that, an overexpression of TRPC6 after a three-week exposure to AgNP, which was consistent with the urinary microalbumin findings.

The other course of AgNP action is DNA damage in young rats observed in our study, which increases the risk of carcinogenesis. However, our study does not suggest a specific mechanism in that respect and calls for further investigation.

Even so, it provides clear evidence that long-term, low-dose (chronic) exposure to silver nanoparticles can cause mild nephrotoxicity and genotoxicity in juvenile rats. The interpretation of our results is limited by the number of animals and dosage range. We still need to find out whether the effects of silver nanoparticles are dose-dependent and if they target organs. In addition, a new animal study is needed to find out if there is a safe dosage (threshold) for AgNPs. New research should also try to answer how ROS production is related to nanoparticle size, shape, surface area, and chemistry (6, 42, 43, 44, 45, 46). This information may greatly help to modify physical and chemical properties of silver nanoparticles to minimise the risks associated with their various applications.
